# Protein Arginine Methyltransferase 1 Is Essential for the Meiosis of Male Germ Cells

**DOI:** 10.3390/ijms22157951

**Published:** 2021-07-26

**Authors:** Sahar Waseem, Sudeep Kumar, Kanghoon Lee, Byoung-Ha Yoon, Mirang Kim, Hail Kim, Keesook Lee

**Affiliations:** 1 School of Biological Sciences and Technology, Chonnam National University, Gwangju 61186, Korea; saharwaseem1@gmail.com (S.W.); sudbiot@gmail.com (S.K.); 2 BioMedical Research Center, Graduate School of Medical Science and Engineering, Korea Advanced Institute of Science and Technology, Daejeon 34141, Korea; cmdr643@kaist.ac.kr; 3 Personalized Genomic Medicine Research Center, Korea Research Institute of Bioscience and Biotechnology, Daejeon 34141, Korea; cogate@kribb.re.kr (B.-H.Y.); mirang@kribb.re.kr (M.K.)

**Keywords:** PRMT1, ADMA, spermatogenesis, meiosis, DNA double strand break repair

## Abstract

Protein arginine methyltransferase 1 (PRMT1) is a major enzyme responsible for the formation of methylarginine in mammalian cells; however, its function in vivo is not well understood due to its early embryonic lethality in null mice exhibiting spontaneous DNA damage, cell cycle delays, and defects in check point activation. Here, we generated germ cell-specific *Prmt1* knock-out (KO) mice to evaluate the function of PRMT1 in spermatogenesis. Our findings demonstrate that PRMT1 is vital for male fertility in mice. Spermatogenesis in *Prmt1* KO mice was arrested at the zygotene-like stage of the first meiotic division due to an elevated number of DNA double-strand breaks (DSBs). There was a loss of methylation in meiotic recombination 11 (MRE11), the key endonuclease in MRE11/RAD50/NBS 1 (MRN) complex, resulting in the accumulation of SPO11 protein in DSBs. The ATM-mediated negative feedback control over SPO11 was lost and, consequently, the repair pathway of DSBs was highly affected in PRMT1 deficient male germ cells. Our findings provide a novel insight into the role of PRMT1-mediated asymmetric demethylation in mouse spermatogenesis.

## 1. Introduction

Among a variety of known post-translational modifications occurring inside mammalian cells, the protein arginine methylation is one of the most common post-translational modifications mediated by the members of the protein arginine methyltransferase (PRMT) family [1,2,3]. Nine members of the PRMT family have been identified in mammalian cells and PRMTs play major roles in pre-mRNA splicing, DNA damage signaling, mRNA translation, cell signaling, and cell fate decision [4,5,6] via the methylation of their histone [7,8] and nonhistone substrates [6]. PRMT type I (PRMT1-4, 6, and 8), type II (PRMT5 and 9), and type III (PRMT7) enzymes are able to generate ω-N^G^, N^G^-asymmetric dimethylarginine (ADMA), ω-N^G^, N^G^-symmetric dimethylarginine (SDMA), and ω-N^G^ -monomethylarginine (MMA), respectively [3].

PRMT1 is the first mammalian PRMT to be identified, which is responsible for the production of 80% of the ADMA in the proteome, including methylation of histone 4 at arginine 3 (H4R3me2a) [9,10]. PRMT2 acts as co-activator for the androgen receptor and estrogen receptor alpha [11,12]. PRMT3 is the only cytosolic member with no known direct epigenetic functions. PRMT4, also known as the co-activator-associated arginine methyltransferase 1 (CARM1), generates H3R17me2a and H3R26me2a marks and works with several transcription factors including p53, nuclear factor-κB, peroxisome proliferator activated receptor gamma, and c-FOS to regulate the target gene expression [3,13]. The newborn *Prmt4* knock-out (KO) mice are smaller than the wildtype (WT) and die shortly after birth [14]. PRMT5 methylates histones before their incorporation into chromatin during embryogenesis and contributes to the maintenance of the pluripotent and undifferentiated state of embryonic stem cells [15,16]; therefore, the PRMT5 null mice die before birth [16]. PRMT6 is exclusively located in the nucleus and its H3R2me2a marks antagonize the activation of H34Kme3 marks, suggesting its role as a transcriptional repressor [17]. PRMT7 is the only PRMT member to produce MMA marks [18] and regulates the expression of pluripotent genes via the microRNA-controlled double-negative feedback loop [19]. Among all the PRMT members, PRMT8 is largely restricted to neurons and is most frequently mutated (over 100 mutations in the coding region) in cancer genomes in a variety of tissues [20,21].

Although PRMT1 whole-body KO mice are embryonically lethal [22], conditional KO studies have revealed the important roles of PRMT1 in different cellular processes. For example, the deletion of PRMT1 from the muscular system and neural progenitors leads to the failure of muscle differentiation and brain demyelination, respectively [23,24]. PRMT1 has been considered a key factor in the epigenetic control and maintenance of the expression levels of genes involved in the determination of the functions and identities of mature β-cells [25]. In a study of the generation of the *Prmt1* null allele in mice, the mouse embryonic fibroblasts (MEFs) lacking PRMT1 exhibited spontaneous DNA damage, cell cycle delays, and check point activation defects [26]. A large number of PRMT1 substrates are known and their preferred methylation sites are arginine that consist of glycine- and glycine-arginine-rich (GAR) sequences [27]. PRMT1 methylates the DNA damage response protein MRE11 [28], which forms a complex with RAD50 and NBS1 that is referred as the MRN complex [29].

Mammalian spermatogenesis is a highly dynamic and complex process that leads to the generation of male gametes from the spermatogonial stem cells through the processes of mitosis and meiosis [30,31]. Spermatogenesis is controlled and synchronized by several epigenetic and post-translational modifications, such as phosphorylation, acetylation, methylation, sumoylation, and ubiquitination [32,33,34,35,36]. Recent studies suggest the essential role of arginine methylation in the reproduction of both male and female mice. During the first wave of spermatogenesis, three PRMT members (PRMT1, PRMT4, and PRMT5) display elevated mRNA expression levels, with the highest expression levels detected in haploid spermatids [37]. PRMT5 acts as a crucial factor for the survival of mouse primordial germ cells [38,39], maintenance of spermatogonial stem cells [40], and in the progression of spermatogenesis as germ cell-specific PRMT5 deletion with *Stra-Cre* resulting in infertility in male mice [41]. PRMT6 and PRMT7 were also found to be directly and indirectly involved in the process of spermatogenesis [42,43].

In this study, we evaluate the function of PRMT1 in spermatogenesis in greater detail by generating male germ cell-specific *Prmt1* KO mice. These male mice were infertile and had atrophic seminiferous tubules due to aberrant meiosis during spermatogenesis. We found that the PRMT1-deficient germ cells had accumulated DNA double strand breaks (DSBs) and attenuated the ataxia telangiectasia-mutated (ATM)-DSB repair pathway due to the loss of the asymmetric dimethylation of MRE11, resulting in a massive loss of germ cells. Our results suggest that PRMT1 regulates meiosis in germ cells by interacting with the ATM pathway of the DSB repair system and is essential for spermatogenesis and male fertility in mice.

## 2. Results

### 2.1. Deletion of Prmt1 in the Testis Results in a Massive Loss of Germ Cells

**Figure 1:**
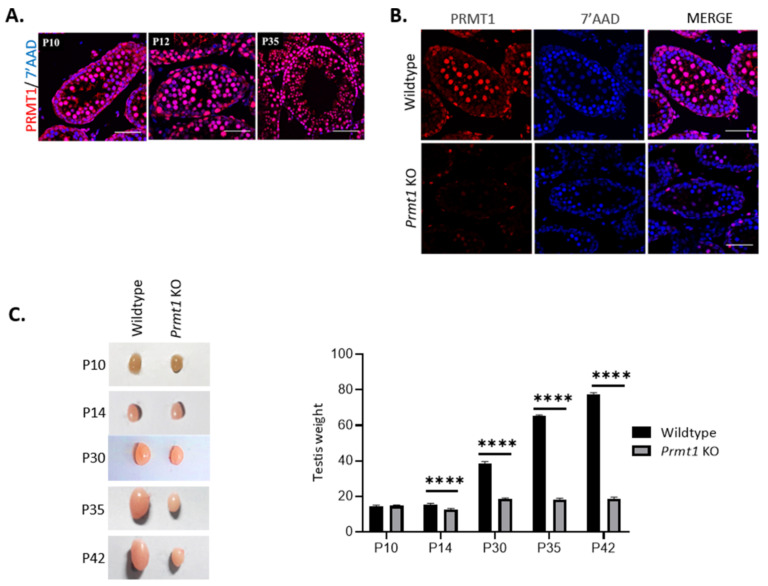

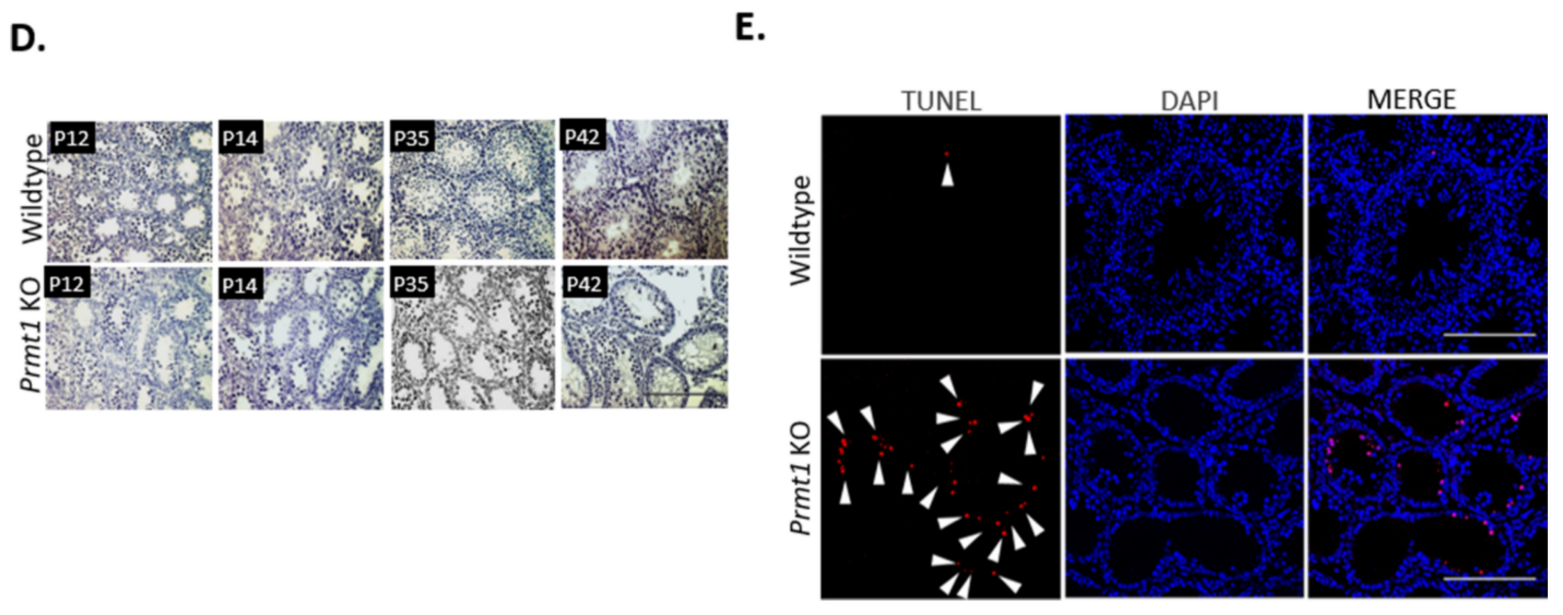
Deletion of the protein arginine methyltransferase 1 (*Prmt1*) in mouse testes results in massive germ cell loss. (**A**) Detection of PRMT1 (pink) expression in wildtype testes on postnatal day 10 (P10), P12, and P35 by immunofluorescence. DNA was stained with 7′-amino actinomycin D (AAD) (blue). Scale bar, 50 μm. (**B**) Confirmation of neurogenin 3 (Ngn3)-Cre-mediated germ cell-specific PRMT1 deletion at P10. PRMT1 (red) colocalized with 7′-AAD (blue). Scale bar, 50 μm. (**C**) Morphological analysis of P10, P14, P30, P35, and P42 testes of the wildtype and *Prmt1* KO mice. Differences in size start on P14, with approximately 70% reduction in the weight of the *Prmt1* KO mouse testis compared with the weight of the control mouse testis. **** *p* < 0.0001. (**D**) Hematoxylin staining of wildtype and *Prmt1* KO testes on P12, P14, and P35. Differences in types and number of germ cells start on P12, and become significantly prominent with an increase in the age of the mice. Most of the seminiferous tubules were atrophic in adult (P35 and P42) *Prmt1* KO testes. Scale bar, 50 µm. (**E**) Immunofluorescence results of the TUNEL (red) analysis of the wildtype and PRMT1 knockout testes on P35. The DNA was stained with DAPI (blue). White arrows indicate apoptotic cells. Scale bar, 50 μm.

To investigate the importance of PRMT1 in spermatogenesis, we specifically knocked out the *Prmt1* gene in germ cells by mating *Prmt1^f^*^/*f*^ mice with neurogenin 3 (Ngn3)-Cre mice that specifically expressed the Cre recombinase enzyme driven by the Ngn3 promoter in germ cells as early as post-natal day 7 (P7) (Figure S1A,B). The *Prmt1^f/f^ Ngn3-Cre* mice had specific deletion of exons 3–4 of *Prmt1*. We examined the expression of PRMT1 in wildtype mouse testes on postnatal day 10 (P10), P12, and P35. PRMT1 is expressed in the nucleus of germ cells at every stage of spermatogenesis and the expression increases with the increase in the number of germ cells during testicular development (Figure 1A). Ngn3 is well expressed in the spermatogonia on P7 [44]. Germ cell-specific *Prmt1* KO mice exhibit negligible expression levels of PRMT1 in germ cells on P10 (Figure 1B). The *Prmt1* KO male mice were found to be completely infertile while the female KO mice were fertile (Table S1). The sizes of the testes of the *Prmt1* KO mice were significantly smaller than that of their control littermates from P14 onwards, with an approximately 70% drop in testes weight at the adult stage (P42) (Figure 1C). Histological analysis of testes revealed that virtual changes, such as differences in the composition of germ cells and increase in the number of atrophic tubules, were observed from P12 onwards. In addition, upon reaching the adult stage (P42) most of seminiferous tubules in *Prmt1* KO were atrophic without germ cells, from haploid spermatids to spermatozoa, suggesting severe problems in the meiosis stage during spermatogenesis (Figure 1D). TUNEL assays displayed significantly higher number of germ cells having apoptotic marks in the *Prmt1* KO testes than their littermate controls (Figure 1E). Collectively, these results indicate that PRMT1 is essential for male fertility in mice, in the absence of which, the germ cells undergo apoptosis before the completion of spermatogenesis and, hence, cannot develop into functional spermatids.

### 2.2. PRMT1 Deficiency Leads to the Reduction in Global Asymmetric Dimethylation as Well as Arginine-3 Dimethylation of Histone H4 in Male Germ Cells

**Figure 2:**
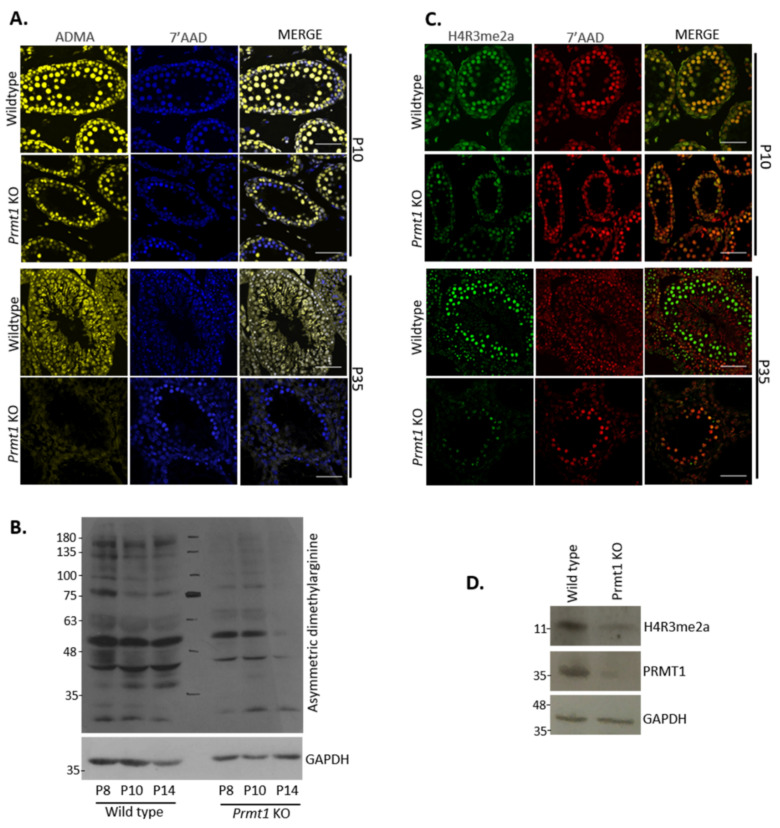
PRMT1 deficiency leads to the reduction of global asymmetric dimethylation as well as asymmetrical dimethylation on arginine-3 of histone H4 (H4R3me2a) in male germ cells. (**A**) Detection of asymmetric dimethylarginine (ADMA) (yellow) mark in wildtype and *Prmt1* KO testes on P10 and P35 by immunofluorescence. DNA was stained with 7′-AAD (blue). Scale bar, 50 μm. (**B**) Western blot analysis to check total ADMA in total protein from wildtype and *Prmt1* KO testes on P8, P10, and P14. GAPDH was used as an endogenous control. (**C**) Detection of H4R3me2a (green) expression in wildtype and *Prmt1* KO testes on P10 and P35 by immunofluorescence. DNA was stained with 7′-AAD (red). Scale bar, 50 μm. (**D**) Western blotting analysis to check H4R3me2a of total protein from wildtype and *Prmt1* KO testes at P20. GAPDH was used as an endogenous control.

As PRMT1 is the predominant PRMT member, we speculated that a difference in the ADMA type of methylation would be observed in the absence of PRMT1, if any other PRMT member did not compensate its function. To address this question, immunofluorescence was performed to check the total ADMA deposits. Testes from P10 and P35 were stained with anti-ADMA antibodies. Strong signals of ADMA were spotted in the germ cells of control testes at P10 and P35. However, significantly less and reduced signals were observed in the germ cells of *Prmt1* KO testes at P10, which further disappeared at the adult stage (P35) (Figure 2A). In addition, Western blotting analysis of whole testis lysates from P8, P10 and P14 also confirmed the presence of highly reduced ADMA signals in the *Prmt1* KO mice compared with the controls (Figure 2B).

We further investigated asymmetric dimethylation of arginine 3 of histone 4 in *Prmt1* KO testes, which is one of the major targets of PRMT1 that is known for the transcriptional regulation of a large number of genes [45]. Immunofluorescence assays were performed with the testes of *Prmt1* KO mice from P10 and P35 by using the anti-H4R3me2a antibody. There was a significant reduction in the levels of H4R3me2a marks in *Prmt1* KO testes, which was further confirmed by western blotting (Figure 2C,D). These findings that the absence of PRMT1 leads to a drastic reduction in the levels of ADMA marks and H4R3me2a methylation indicate that the function of PRMT1 cannot be compensated by other PRMTs in male germ cells in mice.

### 2.3. PRMT1 Deletion Alters the Expression Patterns of Genes Involved in Mouse Spermatogenesis

**Figure 3:**
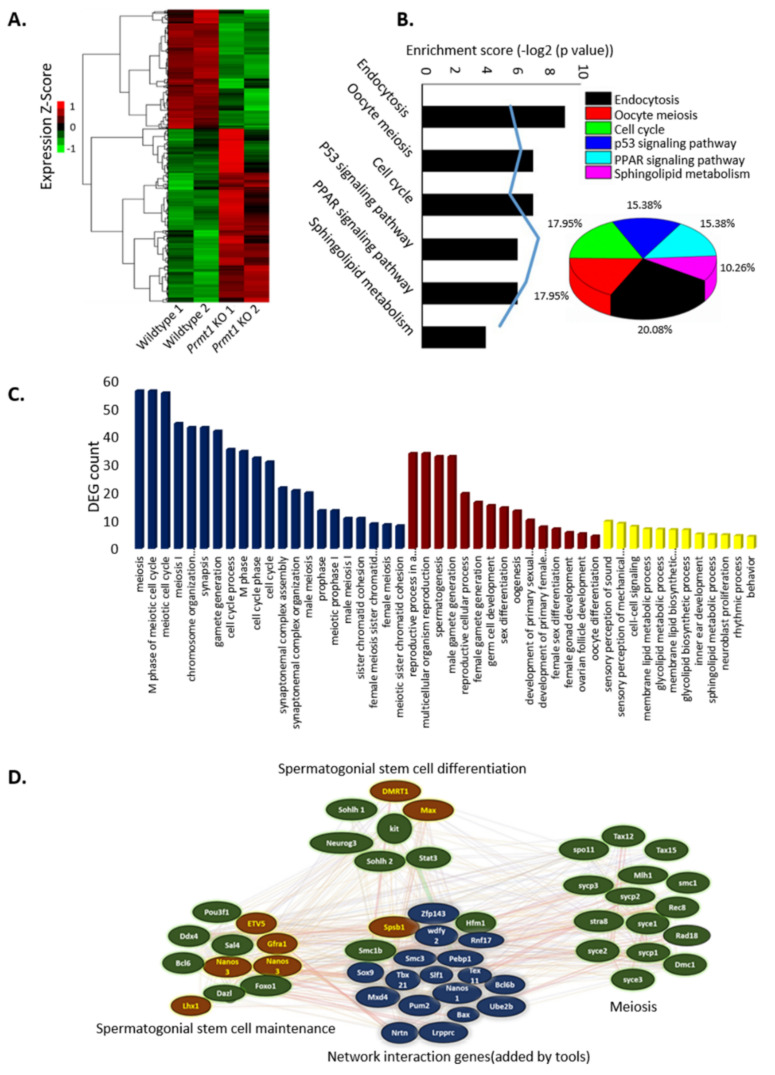
PRMT1 deletion alters the expression pattern of genes involved in mouse spermatogenesis. (**A**) Heatmap showing the differentially expressed genes between the wildtype and *Prmt1* KO testes on P8 and their functional enrichment. Two-fold expression difference and *p* = 0.05 as the cut-off. (**B**) Top-enriched Kyoto Encyclopedia of Genes and Genomes (KEGG) pathways of the downregulated differentially expressed genes (DEGs). Bar represents -log2 P value and blue line represents the DEG count. (**C**) Gene ontology enrichment analysis of biological processes for the downregulated genes between the wildtype and *Prmt1* KO testes. Meiosis-related genes are represented in blue, developmental process genes are in red and miscellaneous in yellow. (**D**) The interaction network of genes involved in spermatogenesis. Upregulated (red), downregulated (green), and unchanged genes (blue).

The above-mentioned results clearly demonstrate that the deletion of germ cell-specific PRMT1 causes a block in the progression of spermatogenesis. Therefore, to systematically illuminate the gene expression changes resulting from *Prmt1* KO in germ cells, we compared the transcriptomes of the WT and *Prmt1* KO testes on P8 using high-throughput RNA sequencing (RNA-seq). We found that a total of 1185 genes were differentially expressed in the *Prmt1* KO testis on P8. Of these, 482 genes were downregulated and 703 genes were upregulated (Figure 3A).

Most of the downregulated genes are those involved in meiosis, a key process in germ cell development. Therefore, we further analyzed these downregulated genes focusing on the genes known to be important for the regulation of spermatogenesis, including the meiotic cell cycle and synaptonemal complex assembly. In the Kyoto Encyclopedia of Genes and Genomes (KEGG) pathway analysis, the downregulated genes were summed up mostly into endocytosis, oocyte meiosis, cell cycle, p53 signaling, PPAR signaling, and sphingolipid metabolism processes (Figure 3B). In addition, the gene ontology enrichment analysis performed under three categories, namely meiosis, developmental process, and miscellaneous, revealed that the highly dysregulated genes belonged to the meiosis category and some others belonged to various developmental processes (Figure 3C). We also mapped the genes involved in the three major stages of spermatogenesis (spermatogonial stem cell maintenance, spermatogonial stem cell differentiation, and meiosis) into an interaction network to illustrate the regulatory relationships. *Prmt1* KO testes showed six downregulated genes [POU class 3 homeobox 1 (*Pou3f1*), DEAD-box helicase 4 (*Ddx4*), B-cell lymphoma 6 (*Bcl6*), deleted in azoospermia-like (*Dazl*), forkhead box O1 (*FOXO1*), and *Sal4*] and five upregulated genes [Ets variant gene 5 (*ETV5*), glial cell derived neurotrophic factor (*GDNF*) receptor alpha 1 (*Gfra1*), nanos C2HC-type zinc finger 3 (*Nanos3*), nanos C2HC-type zinc finger 2 (*Nanos2*), and LIM homeobox 1 (*Lhx1*)] involved in spermatogonial stem cell maintenance, five downregulated genes [spermatogenesis and oogenesis specific basic helix-loop-helix 1 (*Sohlh1*), *Ngn3*, spermatogenesis and oogenesis specific basic helix-loop-helix 2 (*Sohlh2*), and signal transducer and activator of transcription 3 (*Stat3*)] and two upregulated genes [doublesex and mab-3 related transcription factor 1 (*Dmrt1*) and MYC associated factor X (*Max*)] involved in spermatogonial stem cell differentiation, and 14 downregulated meiosis specific genes [*Spo11*, *Tax12*, *Tax15*, MutL homolog 1 (*Mlh1*), structural maintenance of chromosome 1 (*Smc1*), synaptonemal complex protein-3 (*Sycp3*), *Rec8,* stimulated by retinoic acid gene 8 *(Stra8*), synaptonemal complex central element protein 1 *(Syce1)*, synaptonemal complex protein-1 (*Sycp1)*, *DNA meiotic recombinase 1 (DMC1*), *Rad18,* and synaptonemal complex central element protein 3 (*Syce3*)] (Figure 3D). This shows significant disturbances in the inter-regulatory network of spermatogenesis in the absence of PRMT1 enzyme, which also suggests the vital role of PRMT1 in the process of spermatogenesis. Collectively, these data reveal that the deletion of germ cell-specific PRMT1 disturbed the expression of genes related to multiple biological processes, among which the genes involved in the process of meiosis were highly affected.

### 2.4. PRMT1 Deficiency Leads to Aberrant Meiosis in Male Germ Cells

**Figure 4:**
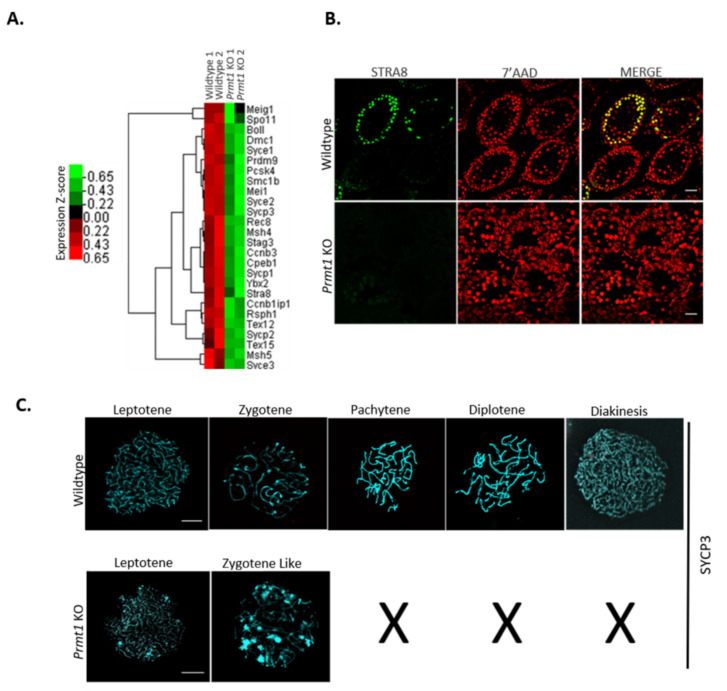
PRMT1 deficiency leads to aberrant meiosis in germ cells of male mice. (**A**) Heatmap showing the differentially expressed meiosis-specific genes between wildtype and *Prmt1* KO testes on P8. Twofold expression difference and *p* = 0.05 as the cut-off. (**B**) Immunofluorescence staining of stimulated by retinoic acid gene 8 (STRA8) in wildtype and *Prmt1* KO testes on P12. Scale bar, 50 μm. (**C**) Immunofluorescence staining to detect the synaptonemal complex protein-3 (SYCP3) on the nuclear surface spreads of spermatocytes from the wildtype and *Prmt1* KO testes on P21. Scale bar, 500 μm.

RNA-seq data of *Prmt1* KO testes revealed that the expression levels of major genes involved in the different stages of meiosis were downregulated in the absence of PRMT1 in male germ cells (Figure 4A), suggesting some severe defects at the time of meiosis in the *Prmt1* KO germ cells. To investigate this possibility, we first checked the expression of STRA8, a marker of meiosis initiation [46,47] on P10 and found that there was negligible expression of STRA8 in *Prmt1* KO testis (Figure 4B). We further exlpored the stage at which meiosis gets arrested in the KO testes by examining the expression of germ cell nuclear antigen 1 (GCNA1) in *Prmt1* KO testes on P14 (Figure S2). GCNA1 is known to be expressed in germ cells at all stages of gametogenesis until the diplotene/dictyate stage of meiosis I [48]. The Human Protein Atlas database suggests that there is high expression of GCNA1 at the leptotene stage, which decreases at the zygotene stage, and very low levels of expression can be seen at the pachytene and further stages of meiosis I. Immunohistochemistry results revealed that germ cells in *Prmt1* KO testis were displaying 70% cells in leptotene (dark) and 30% cells in zygotene (moderate) type of GCNA1 staining, whereas germ cells in the control testis exhibited GCNA1 staining of all types of germ cells (high, moderate, low, and very low) as described in the database. These results suggest the probability of the meiotic arrest of germ cells somewhere at the leptotene or zygotene stage of meiosis I in the absence of PRMT1.

To precisely define the stage of meiotic arrest, we further examined the progression of chromosome synapsis by immunostaining of spermatocye spreads on P21 with anti-SYCP3 antibodies. SYCP3-binding and assembly of meiotic chromosomes leads to their organization into compact structures that are compatible for recombination and crossover formation [49]. Among all SYCP3-positive primary spermatocytes, PRMT1-deficient spermatocytes were found to be either in the leptotene (70%) or zygotene-like stage (30 %), while the control spermatocytes displayed all stages of prophase I, such as leptotene, zygotene, pachytene, diplotene, and diakinesis (Figure 4C). Collectively, these results demonstrate that the deficiency of PRMT1 in male germ cells blocks meiosis at the zygotene-like stage of prophase I.

### 2.5. PRMT1-Deficient Germ Cells Accumulate DNA Double-Strand Breaks

**Figure 5:**
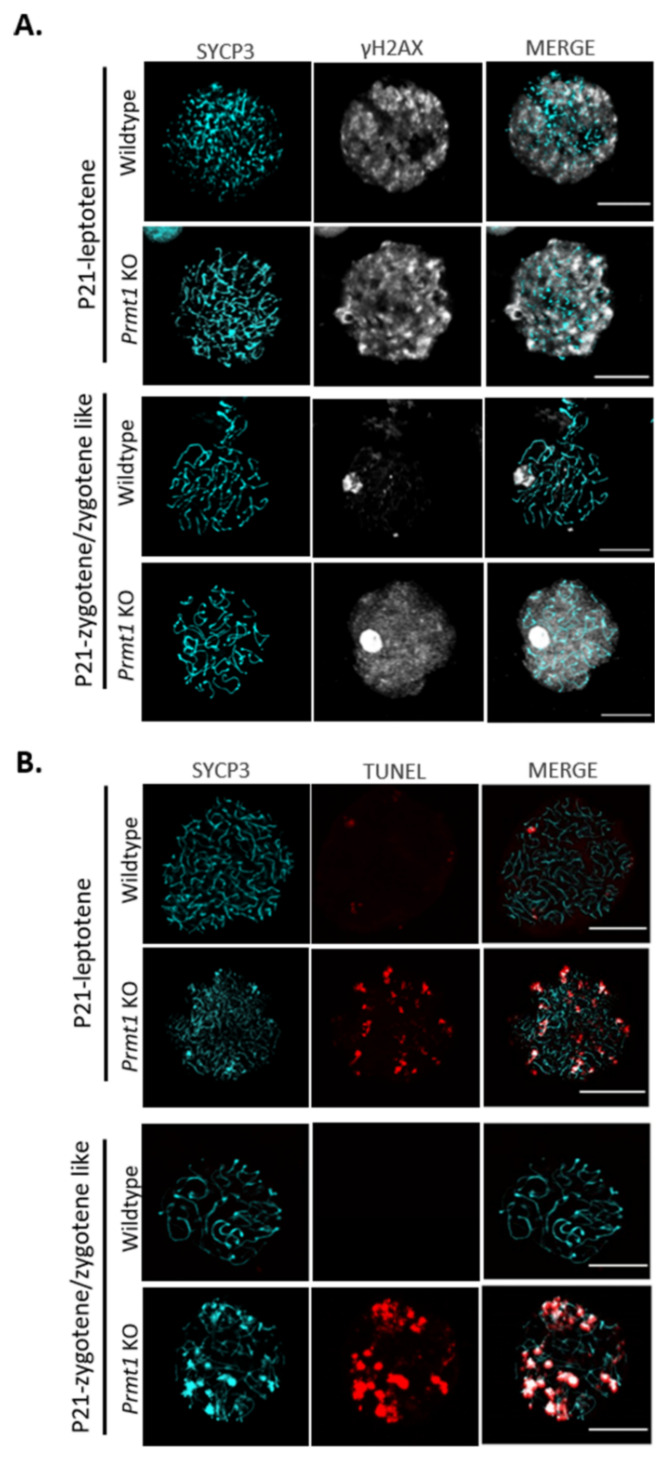
PRMT1-deficient germ cells accumulate DNA double-strand breaks. (**A**) Immunofluorescence staining for the detection of γH2AX and SYCP3 on nuclear surface spreads of leptotene, zygotene, or zygotene-like spermatocytes from wildtype and *Prmt1* KO testes on P21. Scale bar, 200 μm. (**B**) TUNEL staining of the nuclear surface spreads of leptotene, zygotene, or zygotene-like spermatocytes from wildtype and *Prmt1* KO testes on P21. Scale bar, 200 μm. Each experiment was repeated at least three times independently.

The PRMT1-deficient germ cells were not able to cross the zygotene stage of prophase I and a number of genes involved in the repair of DNA DSBs were downregulated in the RNA-seq data (Figure S3A). Therefore, we speculated that this meiotic arrest might be the result of defects in the DNA DSB repair system. To verify this hypothesis, PRMT1-deficient testis was examined for the presence of DSB repair marker protein, γH2AX [50]. The expression of γH2AX is known to be high at the leptotene stage (positive), which decreases around the zygotene stage (partial positive), and the protein is present only in the sex body at the pachytene stage (only sex-body-positive) [51]. Immunofluorescence assay of testis on P14 using anti-γH2AX antibody revealed that only leptotene-type staining (positive) was detected in approximately 90% of seminiferous tubules of *Prmt1* KO testis, while all different types of staining were observed in the seminiferous tubules of WT testis (Figure S3B). We further checked the exact pattern of γH2AX expression in *Prmt1* KO spermatocytes on P21 and found that the leptotene stage exhibited whole chromatin γH2AX staining in both WT and *Prmt1* KO spermatocytes indicating the formation of DSB. However, as the meiosis progressed to the zygotene/zygotene-like stage, γH2AX could still be detected all over the chromatin as well as in the sex body in the *Prmt1* KO zygotene-like spermatocytes, whereas γH2AX foci had disappeared and were only present in the sex bodies in WT spermatocytes, indicating that DSB repair had taken place (Figure 5A).

In addition, TUNEL assays were performed on the nuclear spread of spermatocytes (P21) to confirm general DNA breaks. We found that spermatocytes of *Prmt1* KO testis exhibited significantly higher number of DNA breaks during their leptotene stages than the WT spermatocytes. As meiosis progressed, DNA breaks in the WT zygotene spermatocytes disappeared while *Prmt1* KO zygotene-like spermatocytes were accumulating further DNA breaks compared to those observed in their leptotene stage (Figure 5B). Accumulation of more DNA breaks in *Prmt1* KO spermatocytes could partially reflect the occurrence of DNA fragmentation during apoptosis due to cell cycle arrest. Collectively, these results were consistent with our hypothesis that there were defects in the DNA DSB repair system during the homologous recombination at leptotene stage in the absence of PRMT1.

### 2.6. Loss of Prmt1 Results in Attenuated Atm-Mediated DSB Repair Pathway

**Figure 6:**
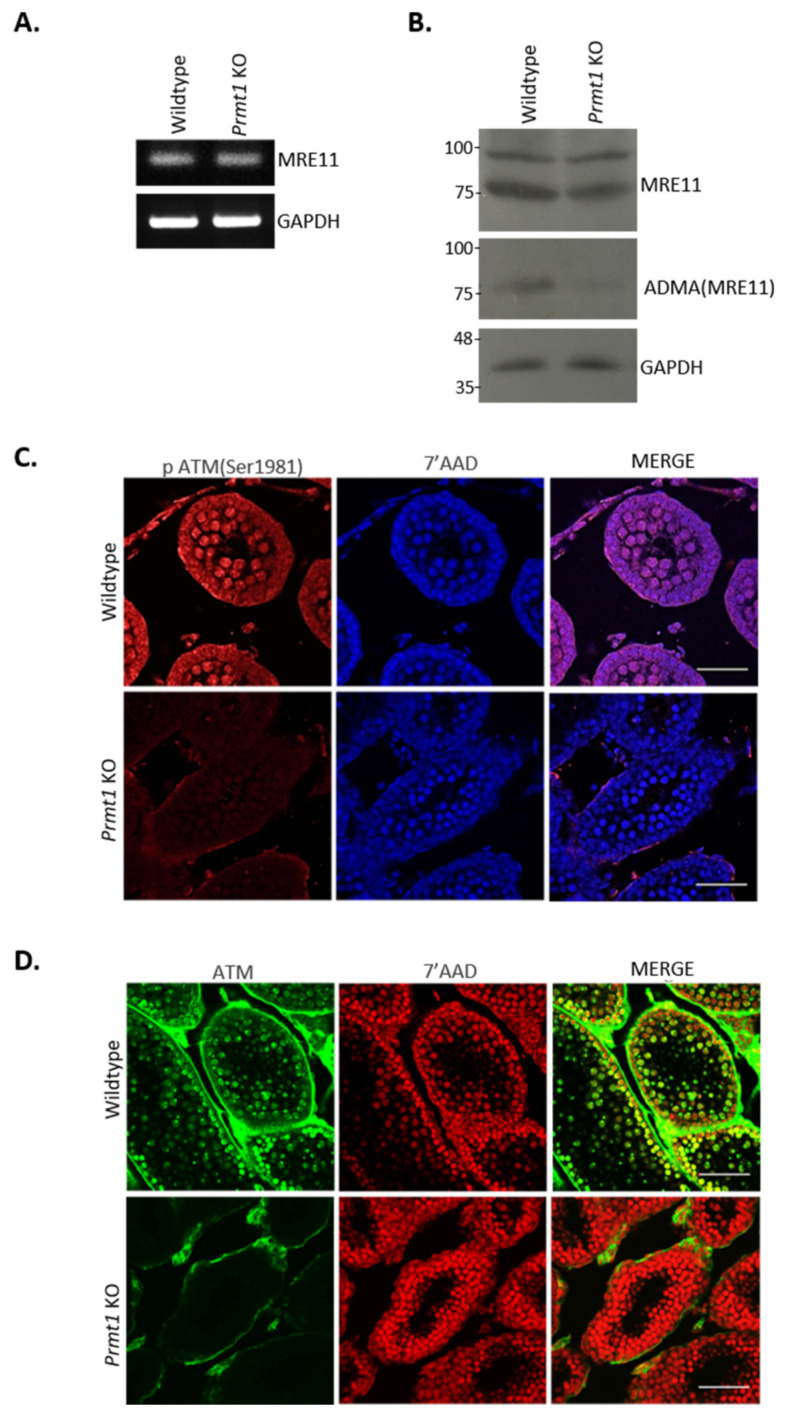

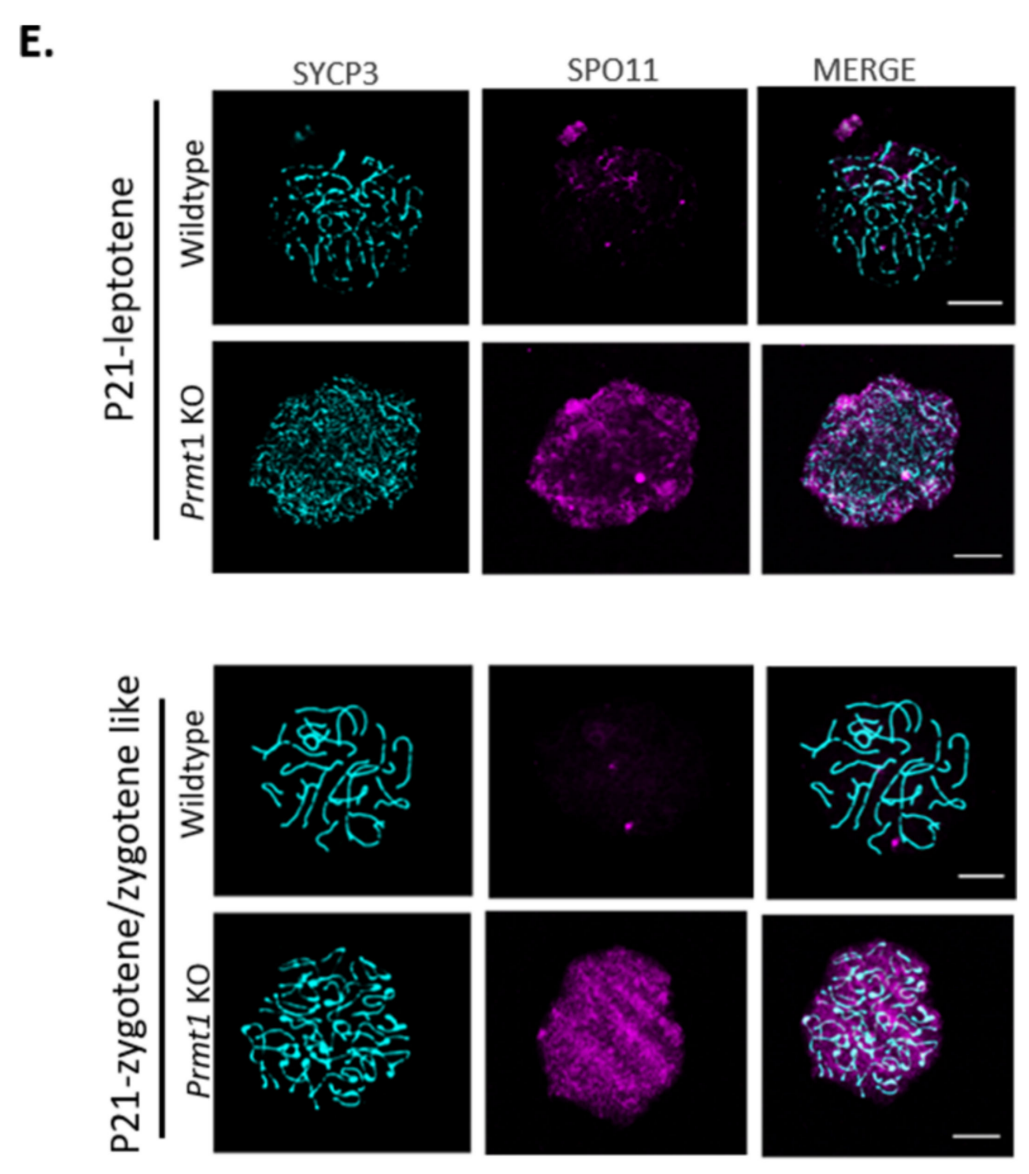
Loss of PRMT1 results in the attenuation in ATM-mediated DSB repair pathway. (**A**) Expression of MRE11 was examined by RT-PCR using cDNA prepared from the total RNA of whole testes on P14. GAPDH was used as an endogenous control. (**B**) Protein expression of MRE11 and ADMA(MRE11) was analyzed by western blotting using whole testis lysates of P14 mice. GAPDH was used as an endogenous control. (**C**) Immunofluorescence staining of pATM (ser1981) (red) in wildtype and *Prmt1* KO testes on P10, co-stained with 7′-AAD (blue). Scale bar, 50 μm. (**D**) Immunofluorescence staining of ATM (green) in wildtype and *Prmt1* KO testes on P21, co- stained with 7′-AAD (red). Scale bar, 50 μm. (**E**) Immunofluorescence staining for the detection of SPO11 and SYCP3 on the nuclear surface spreads of the leptotene, zygotene, and zygotene-like spermatocytes from the wildtype and *Prmt1* KO testes on P21. Scale bar, 200 μm. Each experiment was repeated at least three times independently.

Previous reports signify the importance of ATM signaling for DNA DSB repair during homologous recombination because in the absence of ATM, spermatocytes lack recombination-dependent arrest in response to accumulated unrepaired DSBs and undergo apoptosis [52]. MRE11/RAD50/NBS1 (MRN) complex acts as an important factor in the activation of the ATM pathway [53,54] and the GAR motif of MRE11 is a known substrate of PRMT1. We assumed that the loss of asymmetric dimethylation of MRE11 might have some adverse effect on ATM signaling in the germ cell-specific *Prmt1* KO testes. To test this hypothesis, we first examined the expression levels of MRE11 in *Prmt1* KO testes via PCR and Western blotting analysis. Although normal expression of MRE11 was observed in the absence of PRMT1 (Figure 6A,B), there was a complete loss of ADMA methylation of MRE11 in the *Prmt1* KO testes. These results confirmed the previous finding that MRE11 is arginine-methylated by PRMT1.

In addition, we examined the level of autophosphorylated ATM in *Prmt1* KO testes by performing immunostaining using anti-pATM (ser1981) antibodies. Surprisingly autophosphorylated ATM was completely undetectable in the PRMT1-deficient germ cells (Figure 6C), which was further confirmed by spermatocyte immunostaining (Figure S4A). Further, complete loss of ATM kinase, which is known to be stabilized by autophosphorylation [55], was observed in the PRMT1-deficient germ cells in contrast to their WT controls (Figure 6D and Figure S4B). Therefore, we further examined the downstream of ATM kinase pathway by investigating the expression of p53, a major protein involved in the ATM-mediated DSB repair pathway [56,57], in the testes of P8, P10, and P14 *Prmt1* KO and WT mice. Western blotting analysis of whole testis lysates showed the reduced expression levels of p53 in *Prmt1* KO testes when compared with their control littermates (Figure S4C). We also checked the expression levels of other downstream genes in the ATM-mediated DSB repair pathway, such as *Rad21*, *Rad51*, and *Husb1*. RT-PCR performed with total RNA from P14 testis showed the significantly reduced expression of these genes, especially *Rad21*, in *Prmt1* KO mice when compared to WT mice (Figure S4D).

Our findings suggested that PRMT1 deficiency in germ cells attenuates the ATM-mediated DSB repair pathway during the process of meiosis I. Since ATM kinase is reported to restrain SPO11 activity via a negative feedback loop to suppress further DSB formation [58], there is a possiblity that PRMT1-deficient zygotene-like spermatocytes might be defective in the feedback regulation by ATM kinase, resulting in higher number of DNA DSBs. Thus we analyzed the levels of SPO11 in the PRMT1-deficient spermatocytes and found that SPO11 patches were substantially more prevalent in the leptotene and zygotene-like spermatocytes than their WT counterparts (Figure 6E). All together, these results suggest that the loss of ADMA in MRE11 in the absence of PRMT1 exacerbates the defects in the ATM-mediated DNA DSB repair pathway and SPO11-mediated DSB regulation process during homologus recombination in meiosis I.

## 3. Discussion

In this study, we investigated the role of PRMT1 in the process of spermatogenesis by generating germ cell-specific *Prmt1* KO mice using *Ngn3-Cre*, which is known to be expressed in the spermatogonia on P7. *Prmt1* KO mice showed infertility with approximately 70% reduction in the testis size of adult mice. Transcriptomic analysis revealed that the germ cell-specific deletion of PRMT1 resulted in the downregulation of several meiosis-related genes, suggesting that PRMT1 might play an important role in the regulation of meiosis during spermatogenesis. The disruption of PRMT 1 blocked meiosis at the zygotene stage. Significantly elevated numbers of DSBs were seen in the PRMT1-deficient leptotene and zygotene-like spermatocytes, which suggests that the DNA DSBs during meiosis are not accurately repaired in the PRMT1-deficient germ cells.

Previous reports suggested that PRMT1 might regulate the DNA damage responses via arginine methylation of MRE11 in somatic cells [28]; however, the underlying mechanism of this process still needs to be elucidated. During meiosis, dynamic processes of homologous recombination take place, which involve self-inflicted DNA DSBs as well as their repair after the completion of gene exchange. The process of DNA DSB repair is vital in meiosis because, in the absence of proper DSB repair, cells cannot complete meiosis and subsequently undergo apoptosis [59,60]. In response to DNA DSBs, ATM is rapidly localized to sites of DNA damage with the help of the MRN complex and its kinase activity is activated. The activation of ATM results in autophosphorylation as well as the phosphorylation of a number of downstream substrates, e.g., checkpoint kinase 2 (*Chk2*), p53, and breast cancer 1 (*Brca1*), to initiate checkpoint signaling and promote DSB repair [61,62]. The loss of arginine methylation in MRE11 severely disrupts the endonuclease activity of MRE11 but does not influence its ability to form the MRN complex [28]. The PRMT1-deficient germ cells also lose the capacity to perform ADMA methylation and the endonuclease activity of MRE11. Previously, it was reported that when PRMT1^−/−^ ES cells were exposed to low doses of etoposide (DNA damage inducer), their ability to repair damaged DNA and progress through the cell cycle was slowed down [28]. In a similar manner, meiosis in the PRMT1-deficient germ cells gets stalled around the leptotene and zygotene stages and the cells undergo massive apoptosis as suggested by the TUNEL assay.

Disruption of SPO11 expression results in both male and female infertility in mice [63,64]. The spermatocytes deficient in SPO11 cannot generate DSBs, their homologous chromosomes fail to recombine and synapse, resulting in massive apoptosis in mid-prophase1 [65]. The ATM-deficient mice are also completely infertile as the spermatocytes begin to degenerate soon after their entry into prophase 1 of meiosis, while the oocytes degenerate at a later stage in embryogenesis, prior to dictyate arrest [66]. The number of meiotic DSBs is controlled by ATM and the spermatocytes lacking ATM accumulate at least tenfold more SPO11-oligonucleotide complexes compared to the controls. It is also reported that ATM restrains SPO11 activity via a negative feedback loop, in which the kinase activation by DSBs suppresses further formation of DSBs [58]. In our study, we observed all these phenomena collectively in male germ cells of *Prmt1* KO mice: loss of methylation of MRE11, accumulation of DSBs as evident by high levels of SPO11, and highly reduced level of phosphorylated ATM as well as ATM kinase. These phenomena have separately been reported to accumulate DSBs and halt meiotic recombination along with the degeneration of germ cells by apoptosis. The female KO mice in the current study were normal and fertile. There is a possibility that *Ngn3* is not expressed in female germ cells and thus *Prmt1* is not knocked out or that the function of *Prmt1* is compensated by other members of the PRMT family. Further studies are needed to uncover the detailed mechanism through which PRMT1 regulates the ATM-mediated DSB repair pathway. As per reports, ATM kinase is activated and regulated by the MRN complex [67] and the loss of methylation of MRE11 does not affect its complex formation. However, further studies are needed to understand how the expression of ATM protein and availability of pATM is highly reduced in the PRMT1-deficient germ cells, which will help to unravel the mechanism behind the infertility caused by meiotic arrest and apoptosis in the germ cell-specific PRMT1-deficient mice.

In summary, the results of our study show that PRMT1 plays an important role in the process of spermatogenesis by regulating the methylation of MRE11. The mice with PRMT1-deficient germ cells were infertile with regressive testicular growth. In the absence of PRMT1, an elevated number of meiotic DSBs were accumulated in the germ cells; DNA damage response mechanisms, such as H2AFX phosphorylation and expression of *Rad21* and *Rad51*, were not apparent; and the ATM-mediated DNA damage response was abnormal, resulting in cell cycle arrest at the zygotene-like stage of meiosis I.

## 4. Materials and Methods

### 4.1. Mice

The mice used in this study were the C57 black 6 (C57BL6) strains, which were maintained in a 12-h light/dark cycle and given food and water ad libitum. *Prmt1^f/f^ Ngn3-Cre* mice were generated by mating *Prmt1^f^*^/+^ *Ngn3-Cre* male mice with *Pmrt1^f/f^* female mice. For sample collection, the animals were sacrificed by cervical dislocation. All animal procedures were approved by the Institutional Animal Care and Use Committee (AICUC) of Chonnam National University.

### 4.2. Genotyping of Mice

*Prmt1* KO mice were genotyped using their finger and tail tissues. To prepare the genomic DNA, finger and tail pieces were suspended in 40 μL of digestion buffer [1× modified Gitschier buffer; 67 mM Tris (pH 8.8), 16.6 mM ammonium sulphate ((NH_4_)_2_SO_4_), 6.7 mM magnesium chloride (MgCl_2_), 1% β-mercaptoethanol, and 0.5% Triton X-100] and boiled for 5 min. Samples were allowed to cool down and proteinase K (Roche, Mannheim, Germany) was added to a final concentration of 1 mg/mL. Then the samples were incubated at 55 °C for 1 h. Finally, the samples were boiled for 5 min to denature proteinase K. Thereafter, the samples were cooled down and 1 μL of total genomic DNA was used for PCR. Primers (Table S2) were used to detect the PRMT1 deletion by Ngn3-Cre.

### 4.3. Histological Analysis

For histological analysis: testes from WT and *Prmt1* KO male mice were fixed in Bouin’s solution (Sigma-Aldrich, St Louis, MO, USA) overnight at 4 °C. The testes were dehydrated stepwise through an ethanol series (70%, 80%, 90%, and 100% ethanol) and treated for paraffin embedding. Then, 5-μm sections were cut with a microtome (Leica 820; Leica Biosystems, Nussloch, Germany) and mounted on a glass slide. After dewaxing with Histochoice^®^ clearing agent (VWR life sciences, Solon, OH, USA) and a series of hydration steps (100%, 90%, 80%, and 70% ethanol and PBS), the sections were stained with Mayer’s Hematoxylin (Thermo Fisher Scientific, Hudson, NH, USA) using the standard protocol and imaged with an optical microscope (Carl Ziess exioscope 2, Oberkochen, Germany).

### 4.4. Immunohistochemistry and Immunofluorescence Analysis

For immunohistochemistry, the testes were fixed in Bouin’s solution (Sigma-Aldrich, St Louis, MO, USA) overnight at 4 °C and embedded in paraffin. The slides were processed for immunohistochemistry using the VECTASTAIN ABC HRP Kit (Vector laboratories, Burlingame, CA, USA) according to the manufacturer’s instructions. After antigen retrieval processing, the sections were blocked in the CAS-block solution (Invitrogen, Camarillo, CA, USA) for 2 h and incubated with primary antibodies overnight at 4 °C in a moist chamber. The next day, the samples were incubated with secondary antibodies and then counterstained with Mayer’s Hematoxylin (Thermo Fisher Scientific, Hudson, NH, USA).

For immunofluorescence analysis, 10% formalin solution-fixed and paraffin-embedded mouse testes were cut into 5-μm-thick sections and mounted on glass slides. After rehydration and antigen retrieval (0.01 M citrate buffer, pH 6.0) for 2 min using a microwave oven, the sections were incubated with CAS-block solution for 5 h. After blocking, the sections were probed with primary antibodies overnight at 4 °C in a moist chamber. The next day, the slides were washed with 0.01% Triton X-100 in PBS (PBS-T). The sections were incubated for 3 h with the secondary antibodies at room temperature. The nucleus was counter-stained using 7′-amino actinomycin D (AAD) (Abcam, Cambridge, MA, USA) or DAPI (Sigma, St Louis, MO, USA). The sections were mounted by Invitrogen ProLong Gold Antifade reagent (Life technologies, Eugene, OR, USA). The images were visualized by confocal microscopy (Leica TCS SPE; Leica microsystems, Wetzlar, Germany) and analyzed using the Leica Application Suite (LAS) AF lite 3.2.0. All the antibodies used have been listed in Table S3.

### 4.5. TUNEL Analysis

The TUNEL assay was performed using the TUNEL Assay Kit–In situ BrdU-Red DNA Fragmentation (ab66110; Abcam, Cambridge, MA, USA) according to the manufacturer’s instructions. DNA was counterstained with DAPI (Sigma, St Louis, MO, USA) for 10 min 25 °C and washed in PBS. The sections were mounted using the Invitrogen ProLong Gold Antifade reagent with DAPI (Life technologies, Eugene, OR, USA). The images were visualized by confocal microscopy (Leica TCS SPE; Leica microsystems, Wetzlar, Germany) and analyzed using the Leica LAS AF lite 3.2.0 Software.

### 4.6. Spermatocyte Preparation and Immunofluorescence Analysis

The meiotic chromosome spreads were prepared as described in a previous study [68], using a solution mixture of 1% paraformaldehyde (PFA) and 0.15% Triton X-100, with pH adjusted to 9.2 with 1 N HCl or 1 N NaOH, as the fixative. The slides were incubated with primary antibodies at 4 °C for 2 h and with secondary antibodies at room temperature for 1 h. The chromosome spreads were mounted in VECTASHIELD Antifade Mounting Medium with DAP (Vector Laboratories, Burlingame, CA, USA).

### 4.7. TUNEL Analysis of Chromosome Spreads

The TUNEL assay was performed using the TUNEL Assay Kit-BrdU-Red (ab66110; Abcam, Cambridge, MA, USA) according to the manufacturer’s instructions but with some modifications according to experimental conditions. Briefly, after treating with primary anti-Sycp3 antibody for 3 h at 4 °C, the spermatocytes were labeled with rTdT reaction mix for 1 h at 37 °C. After washing in PBS, the sections were incubated with anti-BrdU antibody for 30 min at room temperature and washed in PBS. After washing, the slides were incubated with Alexa Fluor 488 Goat Anti-Rabbit Antibody (A11034; ThermoFisher Scientific, Rockford, IL, USA) for 30 min at room temperature. Then, the slides were mounted with VECTASHIELD Antifade Mounting Medium with DAP (Vector Laboratories, Burlingame, CA, USA). The images were visualized by confocal microscopy (Leica TCS SPE, Leica Microsystems, Wetzlar, Germany) and analyzed using the Leica LAS AF Software lite 3.2.0.

### 4.8. RNA Extraction, RT-PCR, and Quantitative Reverse Transcription Polymerase Chain Reaction (qRT-PCR)

Total RNA was extracted with TRI reagent (Molecular research center, Cincinnati, OH, USA) from whole WT and *Prmt1* KO testes. Then, 2 μg of total RNA was reverse-transcribed into cDNA using the Moloney Murine Leukemia Virus (M-MLV) Reverse Transcriptase Kit (Promega Corporation, Fitchburg, WI, USA) and RT-PCR was performed. Further, qPCR was performed using the StepOnePlus Real-Time PCR System (Applied Biosystems, Carlsbad, CA, USA) and TOPreal^TM^ qPCR SYBR PreMix (Enzynomics, Daejeon, Republic of Korea) according to the manufacturer’s instructions. Relative gene expression was examined using the 2^−ΔΔ*CT*^method with GAPDH as an internal control. At least three independent experiments were performed.

### 4.9. RNA Sequencing

RNA was extracted from testes from P8 mice (WT and *Prmt1* KO) using the TRIzol reagent (#15596018, Life Technologies, Carlsbad, CA, USA) and the quality of the extracted total RNA samples was checked with RNA 6000 Nano Kit (#5067-1511, Agilent, Santa Clara, CA, USA) on an Agilent 2100 bioanalyzer, confirming RIN values were above 7. The RNA-seq library was prepared using a TruSeq RNA Sample Prep Kit (Illumina, SanDiego, CA, USA), and sequencing was performed using Illumina HiSeq 3000 platform to generate 100-bp paired-end reads. Genes having *p* < 0.5 were used for the differentially expressed gene analysis.

### 4.10. RNA-Seq Data Analysis

FastQC (FastQC v0.11.3) [69] was used to filter the low-quality sequencing reads. Then, the reads were mapped to mouse genome build mm10 using TopHat (TopHat v2.0.11) [70] with the default parameters. DESeq (DESeq v3.1) [71] was used to select the differentially expressed genes (DEGs) (fold-change > 2 and *p*-value < 0.05). Hierarchical clustering was performed to characterize the overall expression patterns of the DEGs using R statistical language (R version 3.3.1) [72] with the Euclidean distance and complete linkage method. The interaction relationship of genes was constructed using GeneMANIA [73]. All the sequence data were deposited to the Gene Expression Omnibus and accessible through GEO accession number GSE176443.

### 4.11. Quantification and Statistical Analysis

Statistical parameters (e.g., the *n* value), statistical analysis results, statistical significance, and scale bar are all reported in the figure description. To identify significant differences, the data were analyzed using GraphPad Prism v.5.0 (GraphPad software, San Diego, CA, USA). Statistical significance was calculated by Student’s *t*-test. A value of *p* < 0.05 was considered significant for all statistical analyses.

## Data Availability

The sequence data were deposited to the Gene Expression Omnibus and accessible through GEO accession number GSE176443.

## Supplementary Materials

The following are available online at https://www.mdpi.com/article/10.3390/ijms22157951/s1.

## Abbreviations

7AAD: 7′-amino actinomycin D; ADMA: asymmetric dimethylarginine; ATM: ataxia telangiectasia-mutated; CARM1: coactivator-associated arginine methyltransferase 1; DEGs: differentially expressed genes; DSB: double strand breaks; GCNA1: germ cell nuclear antigen 1; GAR: glycine-arginine rich; KEGG: Kyoto Encyclopedia of Genes and Genomes; MEFs: mouse embryonic fibroblasts; MMA: monomethyl arginine; MRE11: meiotic recombination 11; Ngn3: neurogenin; NBS1: nibrin 1; PRMT1: protein arginine methyltransferase 1; qRT-PCR: quantitative reverse transcription polymerase chain reaction SSCs: spermatogonial stem cells; STRA8: stimulated by retinoic acid gene 8; SYCP3: synaptonemal complex protein 3.
