# Supplementary material for: Protein Arginine Methyltransferase 1 Is Essential for the Meiosis of Male Germ Cells
Source: Int J Mol Sci. 2021 Jul 26;22(15):7951. doi: 10.3390/ijms22157951 (PMC8348183; doi:10.3390/ijms22157951)
Supplement: Supplementary file 1 [file ijms-22-07951-s001.zip › ijms-1308770-supplementary.pdf]

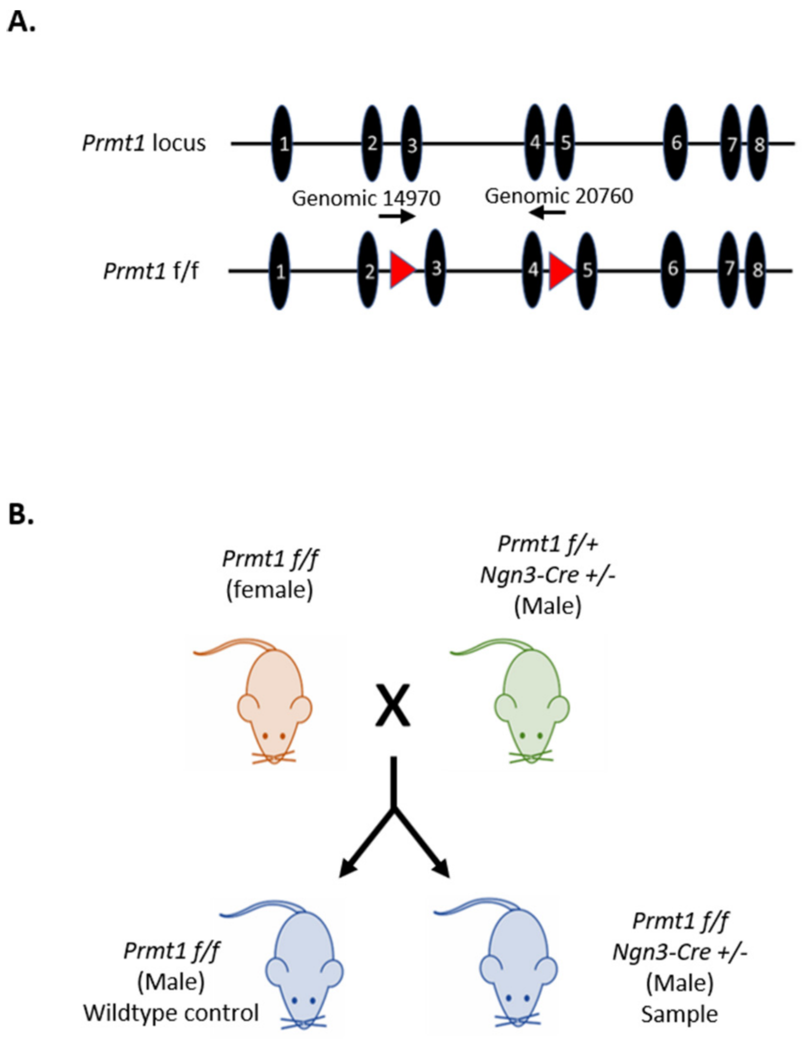

Figure S1. Generation of PRMT1 knockout mice. (A) Schematic representation of the wildtype [protein arginine methyltransferase 1 (PRMT1)] and floxed (2 loxP; *Prmt1*$^{f/f}$) loci. The exons are represented by the black oval shapes. Red triangles denote the loxP sites. Deleted exons are 3 and 4, in which exon 3 domain is conserved for S-adenosyl methionine binding, and the arrows denote the primers (named genomic 14970 and genomic 20760) used for PCR analysis. **(B)** Using the Cre/loxP recombination system, germ cell-specific *Prmt1* KO mice were generated by crossing *Prmt1*$^{f/f}$ female mice with *Prmt1*$^{f/+}$/*Ngn3-Cre* male. *Prmt1*$^{f/f}$/*Ngn3-cre* male mice were used as an experiment model.

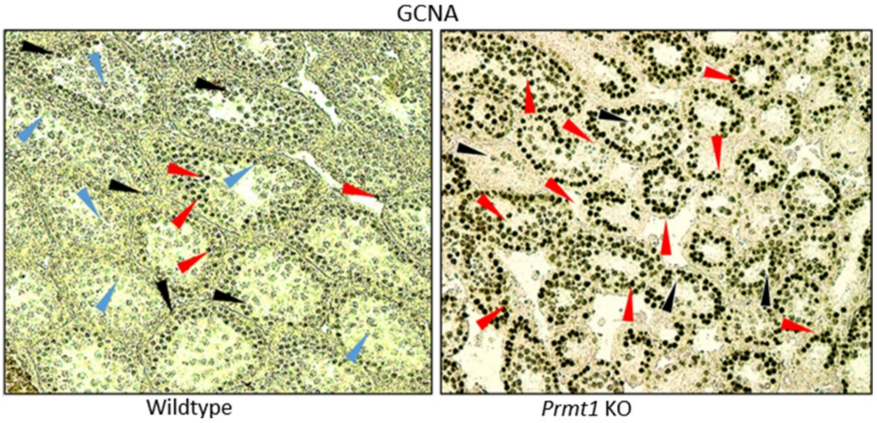

Figure S2. Staining of germ cells in the wildtype and PRMT1 knockout mice. Immunohistochemical staining of germ cell nuclear antigen 1 (GCNA1) was performed with the wildtype and PRMT1 knockout testes on P14. Scale bar, 50 μm. Arrows indicate spermatocytes at different stages of spermatogenesis: black (leptotene), red (zygotene), and blue (pachytene).

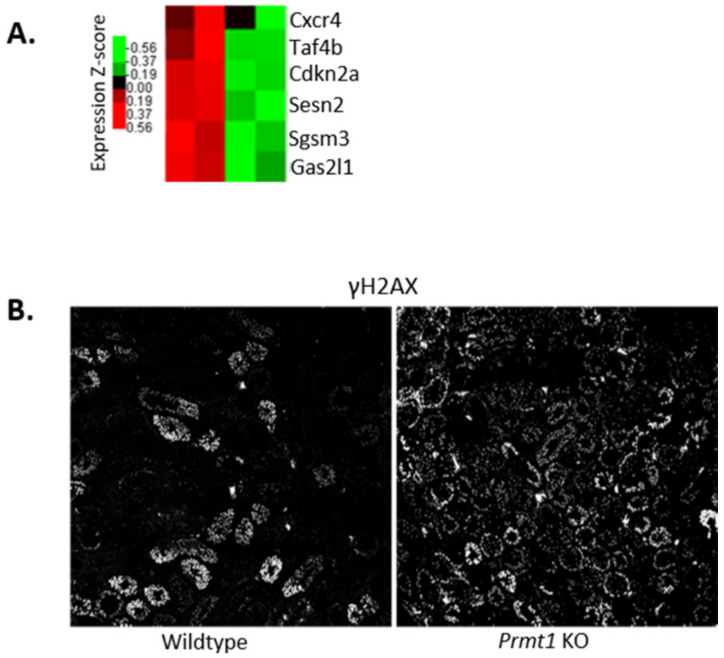

Figure S3. PRMT1-deficient germ cells accumulate DNA double-strand breaks. (A) Heat map showing the differentially expressed genes involved in the DNA- double strand break (DSB) repair pathway in the wildtype and *Prmt1* KO testes on P8. **(B)** Immunoflorescence staining of γH2AX in wildtype and *Prmt1* KO testes on P14. Scale bar, 50 μm.

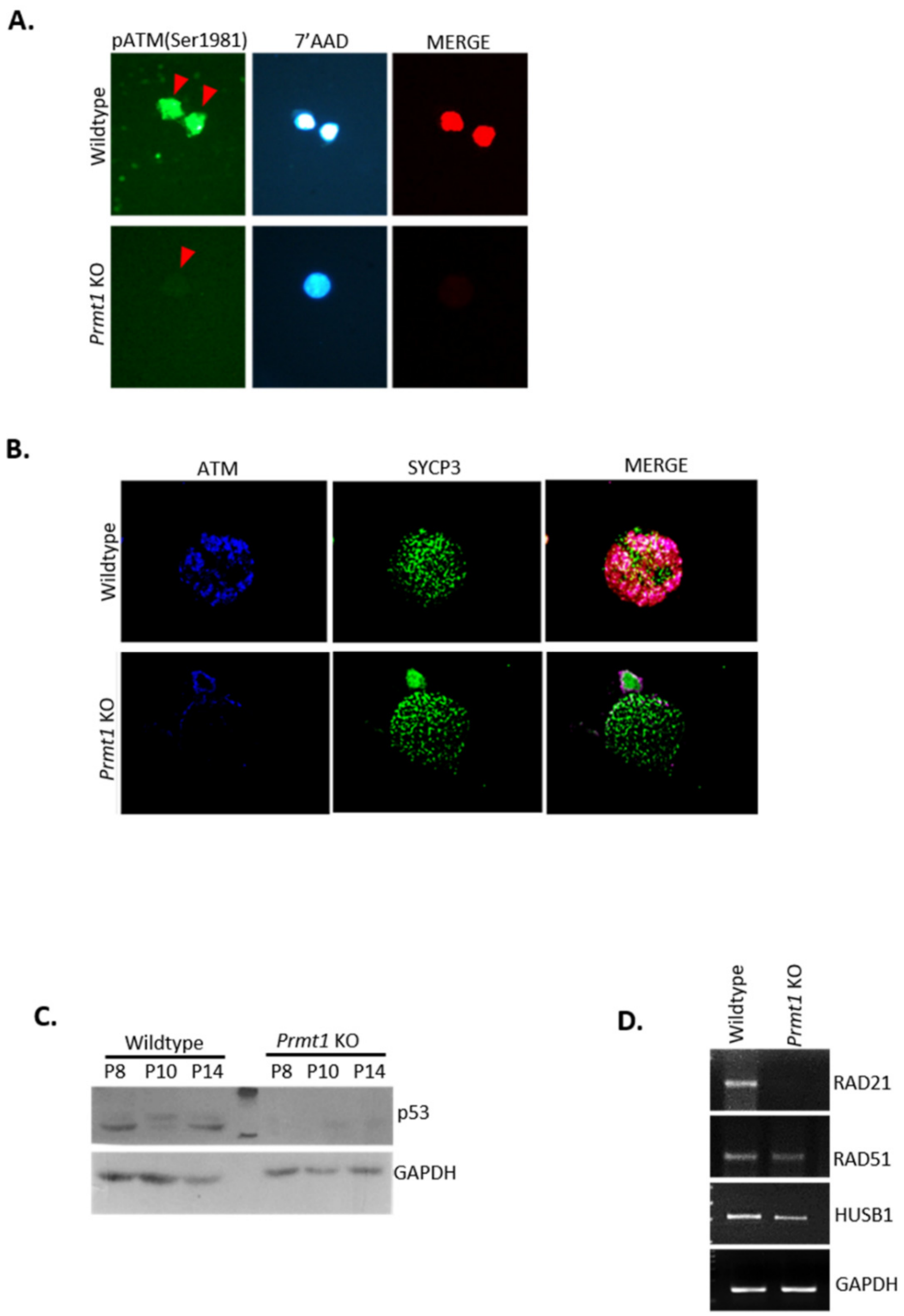

Figure S4. Loss of PRMT1 results in the attenuation of the ATM-mediated DSB repair pathway. (A) Immunofluorescence staining for the detection of pATM (ser1981) (green) in spermatocytes from wildtype and *Prmt1* KO testes, which were co-stained with 7′AAD (blue). **(B)** Immunofluorescence staining for the detection of ATM and synaptonemal complex protein-3 (SYCP3) on the nuclear surface spreads of leptotene spermatocytes from wildtype and *Prmt1* KO testes at P21. Scale bar, 200 µm (**C**) Protein expression levels of p53 were checked by western blotting using whole testes lysates of P8, P10 and P14 mice. GAPDH was used as an endogenous control. (**D**) Gene expression levels of *Rad21*, *Rad51*, and *Husb1* were analyzed by RT-PCR using cDNA prepared from the total RNA of whole testes on P14. GAPDH was used as an endogenous control.

Table S1: Fertility of *Prmt1* KO mice

Mating genotype		Average number of pups /litters
Male	Female	
Wildtype x	Wildtype	7.5 ± 0.43
PRMT F/F x	PRMT1 F/F Ngn3-cre	7.8 ± 0.41
PRMT1 F/F Ngn3-Cre x	PRMT F/F	0

The number of pups from breeding wild-type and homozygous PRMT1 knockout mice is presented as mean +/- SEM (n=5 for each group)

Table S2: List of Primers.

Genotyping primers

Primer name	Sequence (5′–3′)
PRMT1 f/f (F)	GTGCTTGCCATACAAGAGATCC
PRMT1 f/f (R)	ACAGCCGAGTAGCAAGGAGG
NGN3- cre (F)	GATGCAACGAGTGATGAG
NGN3- cre (R)	ACAGCCGAGTAGCAAGGAGG

PCR and qRT-PCR primers

Primer name	Sequence (5′–3′)
Ckit (F)	TCAATCGAGTGTGATGGGAAA

Ckit (R)	GGTGACTTGTTTCAGGCACA
GAPDH (F)	ATCACCATCTTCCAGGAGCGAG
GAPDH (R)	GAGATGATGACCCTTTTGGCTCC
HUSB1 (F)	TACCACCGAGGTTTTGAGGC
HUSB1 (R)	GGTGCTGCTGACCTGAATGA
MRE11(F)	AGGTTTCTGGGAGCGGTTTT
MRE11(R)	CGTCAAGTGGATCTGTGGGG
NGN3 (F)	GAGAACTAGGATGGCGCCTC
NGN3(R)	GCAGTCCCTAGGTATGAGAGTG
PLZF (F)	CCCAGTTCTCAAAGGAGGATG
PLZF (R)	TTCCCA CACAGCAGACAGAAG
RAD 21 (F)	GAGGCTACACCGCACAAGG
RAD 21 (R)	AGAAGAAACCTGGATCTCGGC
RAD 51 (F)	AACCTGACAGAGGAGCAACG
RAD 51 (R)	GCACTTGCTTCAAGCTGCAT

Table S3: List of antibodies.

Antibody	Catalogue number	Company
Rabbit anti-STRA8	ab49602	Abcam
Rabbit anti-SCP3	ab15093	Abcam
Rabbit anti - MRE 11	ab33125	Abcam

Rabbit anti-PRMT1	ab73246	Abcam
Mouse anti-ATM	ab78	Abcam
Mouse anti- p-Histone H2A.X(Ser 139)	Sc-517348	Santa Cruz Biotechnology
Mouse anti- spo11	Sc-377161	Santa Cruz Biotechnology
Rabbit anti - GAPDH	Sc-25778	Santa Cruz Biotechnology
Rabbit anti- P53	Sc-6243	Santa Cruz Biotechnology
Mouse anti-PRMT1	Sc-59648	Santa Cruz Biotechnology
Rabbit anti-Adme-R	#13522	Cell signaling Technology
Rabbit anti-Phospho -ATM (Ser1981)	D6H9	Cell signaling Technology
Anti -Rabbit IgG	65-6140	Thermo Fisher Scientific
Anti-Mouse IgM	Sc-2064	Santa Cruz Biotechnology
Anti-Mouse IgG	Sc-516102	Santa Cruz Biotechnology
Anti-Mouse Alexa flour 488	A10680	Thermo Fisher Scientific
Anti-Rabbit Alexa flour 488	A11034	Thermo Fisher Scientific
Anti-Mouse Alexa flour 594	A11012	Thermo Fisher Scientific
